# Influence of Vitamin D supplementation on reproductive outcomes of infertile patients: a systematic review and meta-analysis

**DOI:** 10.1186/s12958-023-01068-8

**Published:** 2023-02-03

**Authors:** Xiangqian Meng, Jiayao Zhang, Qi Wan, Jihua Huang, Tingting Han, Ting Qu, Lin-lin Yu

**Affiliations:** 1 Chengdu Xi’nan Gynecological Hospital Co. LTD, Chengdu, 610000 Sichuan China; 2 West China School of Basic Medical Sciences & Forensic Medicine, Sichuan University, Chengdu, 610000 Sichuan China; 3 Chengdu Jinxin Research Institute for Reproductive Medicine and Genetics, Chengdu, 610000 Sichuan China; 4 Chengdu Women’s and Children’s Central Hospital, School of Medicine, University of Electronic Science and Technology of China, Chengdu, 610000 Sichuan China

**Keywords:** Vitamin D, Supplementation, Reproductive outcomes, Infertile women, Clinical pregnancy rate

## Abstract

**Background:**

Low vitamin D status has been associated with an increased risk for infertility. Recent evidence regarding the efficacy of vitamin D supplementation in improving reproductive outcomes is inconsistent. Therefore, this systematic review was conducted to investigate whether vitamin D supplementation could improve the reproductive outcomes of infertile patients and evaluate how the parameters of vitamin D supplementation affected the clinical pregnancy rate.

**Methods:**

We searched seven electronic databases (CNKI, Cqvip, Wanfang, PubMed, Medline, Embase, and Cochrane Library) up to March 2022. Randomized and cohort studies were collected to assess the reproductive outcomes difference between the intervention (vitamin D) vs. the control (placebo or none). Mantel-Haenszel random effects models were used. Effects were reported as odds ratio (OR) and their 95% confidence interval (CI). PROSPERO database registration number: CRD42022304018.

**Results:**

Twelve eligible studies (*n* = 2352) were included: 9 randomized controlled trials (RCTs, *n* = 1677) and 3 cohort studies (*n* = 675). Pooled results indicated that infertile women treated with vitamin D had a significantly increased clinical pregnancy rate compared with the control group (OR: 1.70, 95% CI: 1.24–2.34; *I*^2^ = 63%, *P* = 0.001). However, the implantation, biochemical pregnancy, miscarriage, and multiple pregnancy rates had no significant difference (OR: 1.86, 95% CI: 1.00–3.47; *I*^2^ = 85%, *P* = 0.05; OR: 1.49; 0.98–2.26; *I*^2^ = 63%, *P* = 0.06; OR: 0.98, 95% CI: 0.63–1.53; *I*^2^ = 0%, *P* = 0.94 and OR: 3.64, 95% CI: 0.58–11.98; *I*^2^ = 68%, *P* = 0.21). The improvement of clinical pregnancy rate in the intervention group was influenced by the vitamin D level of patients, drug type, the total vitamin D dosage, the duration, administration frequency, and daily dosage of vitamin D supplementation. The infertile women (vitamin D level < 30 ng/mL) treated with the multicomponent drugs including vitamin D (10,000–50,000 IU or 50,000–500,000 IU), or got vitamin D 1000–10,000 IU daily, lasting for 30–60 days could achieve better pregnancy outcome.

**Conclusion:**

To the best of our knowledge, this is the first meta-analysis systematically investigated that moderate daily dosing of vitamin D supplementation could improve the clinical pregnancy rate of infertile women and reported the effects of vitamin D supplementation parameters on pregnancy outcomes. A larger sample size and high-quality RCTs are necessary to optimize the parameters of vitamin D supplementation to help more infertile patients benefit from this therapy.

**Supplementary Information:**

The online version contains supplementary material available at 10.1186/s12958-023-01068-8.

## Introduction

Infertility is a widespread health problem across the world. Approximately 9.3–16.7% of the females of child-bearing age suffered from infertility [1, 2]. In recent years, an increasing number of infertile women seek assistance from assisted reproductive techniques (ARTs) [3]. However, the efficacy of improvement in ARTs slowed down recently [4]. It is still necessary to improve the effectiveness of ARTs. Vitamin D, a steroid hormone, has five compounds in which vitamin D_2_ (ergocalciferol) and vitamin D_3_ (cholecalciferol) are vital members associated with reproductive health [5]. Previous research found that 1α-hydroxylase (vitamin D enzymes) and vitamin D receptors were expressed in human first-trimester and decidua [6, 7]. Vitamin D receptors and 1,25(OH)_2_D_3_ regulated the transcription of HOXA10 which was the key target gene associated with implantation [6–8]. Accumulating evidence from prospective random and cohort observational studies proposed that vitamin D insufficiency or deficiency was related to infertility [9]. It is proposed that vitamin D status might influence initial embryo implantation by regulating the immunology cells (natural killer cells, dendritic cells, macrophages, and T cells) in uterine and decidua tissue [6, 7]. However, recent interest focused on the association between vitamin D levels and ART outcomes, but not on the influence of vitamin D supplementation on reproduction [9]. The animal experiment found the injection of vitamin D_3_ could induce the decidualization of rat endometrial cells [10]. In human clinical trials, some studies found vitamin D supplementation improved the reproductive outcomes of infertile women [11, 12], but other research showed the failed influence of vitamin D treatment on pregnancy outcomes [13, 14]. Whether vitamin D supplements could contribute to successful ARTs outcomes of infertile women was still uncertain. Similarly, the dosage and duration of vitamin D supplementation varied greatly in the previous reports [13, 15]. The high concentration of serum vitamin D could result in hypervitaminosis D (vitamin D poisoning) which was associated with nausea, vomiting, weakness, disturbed digestion, and elevated blood and tissue calcium levels [16–18]. Considering appropriate vitamin D supplementation for overall health benefits, it is of great significance to investigate the fertility effect of parameters of vitamin D supplementation.

There are lack of conclusive results and a comprehensive review regarding the actual fertility benefits of vitamin D supplementation and the potential effects of its parameters. Therefore, in this systematic review and meta-analysis, our purpose was to evaluate whether vitamin D supplementation could influence the reproductive outcomes of infertile women, and provide practical guidance on the parameters of vitamin D supplementation to ensure infertile patients could receive proper treatment and improve the treatment effectiveness for future trials.

## Methods

This systematic review and meta-analysis followed the Preferred Reporting Items for Systematic Reviews and Meta-Analyses (PRISMA) guidelines. The protocol of this study was prospectively registered with the registration number CRD42022304018 at PROSPERO. The institutional review board approval was not required because all data were published previously.

### Search strategy

English-language databases PubMed, Medline, Embase, and Cochrane Library and Chinese-language databases CNKI, Cqvip, and Wanfang were searched. The search strategy was devised for each outcome (Supplemental Search strategy, available online). Searches time was restricted to studies published up to March 2022. References from the selected articles, including relevant review papers, were reviewed to identify all relevant studies. Conference abstracts and prospective trial registries were also searched for relevant items.

### Inclusion and exclusion criteria

Data were carefully extracted by 2 investigators independently. Any inconsistent opinions were resolved by discussion or with the help of a further investigator. The infertile women undergoing ART (IVF, ICSI, fresh embryo transfer, and frozen embryo transfer) who had vitamin D supplementation were recruited. Study characteristics [authors’ last name(s), year of publication, country, and population (number of cases and controls)], specific details about the interventions and reproductive outcome measures (implantation rate, biochemical pregnancy rate, clinical pregnancy rate, miscarriage rate, and multiple pregnancy rate) were recorded and summarized. Exclusion criteria were: (1) reviews and case reports; (2) duplicate publications; (3) data were not available or could not be extracted for the study groups; and (4) no appropriate case or control group.

### Quality assessment

Quality assessment was evaluated by 2 investigators independently. Any inconsistent opinions were arbitrated by a third investigator. The risk of bias for RCTs was evaluated using Cochrane’s tool. The quality score of cohort studies was assessed using Newcastle-Ottawa Scale. The quality scores of studies ranged from 0 to 9 points and included three aspects: selection, comparability, and exposure.

### Statistical Analysis

The extracted data were analyzed with Review Manager 5.3 software (Cochrane Collaboration, Oxford, U.K.). The Mantel-Haenszel method random-effects models were used for meta-analysis. The effect sizes were expressed as odds ratios (ORs) and calculated using their 95% confidence intervals (CIs). Summary ORs and 95% CIs were assessed graphically with forest plots. The Heterogeneity was quantified using the *I*^2^ value. To examine the potential heterogeneity sources, subgroup meta-analyses were performed according to the vitamin D level of patients, drug type, the total vitamin D dosage, and the duration, administration frequency, and daily dosage of vitamin D supplementation. Publication bias was evaluated using a funnel plot. To evaluate whether there was any study affecting the stability of the results, STATA 17.0 software was used for the sensitivity analysis (leave one out). A *P*-value <0.05 was considered statistically significant.

## Results

**Fig. 1:**
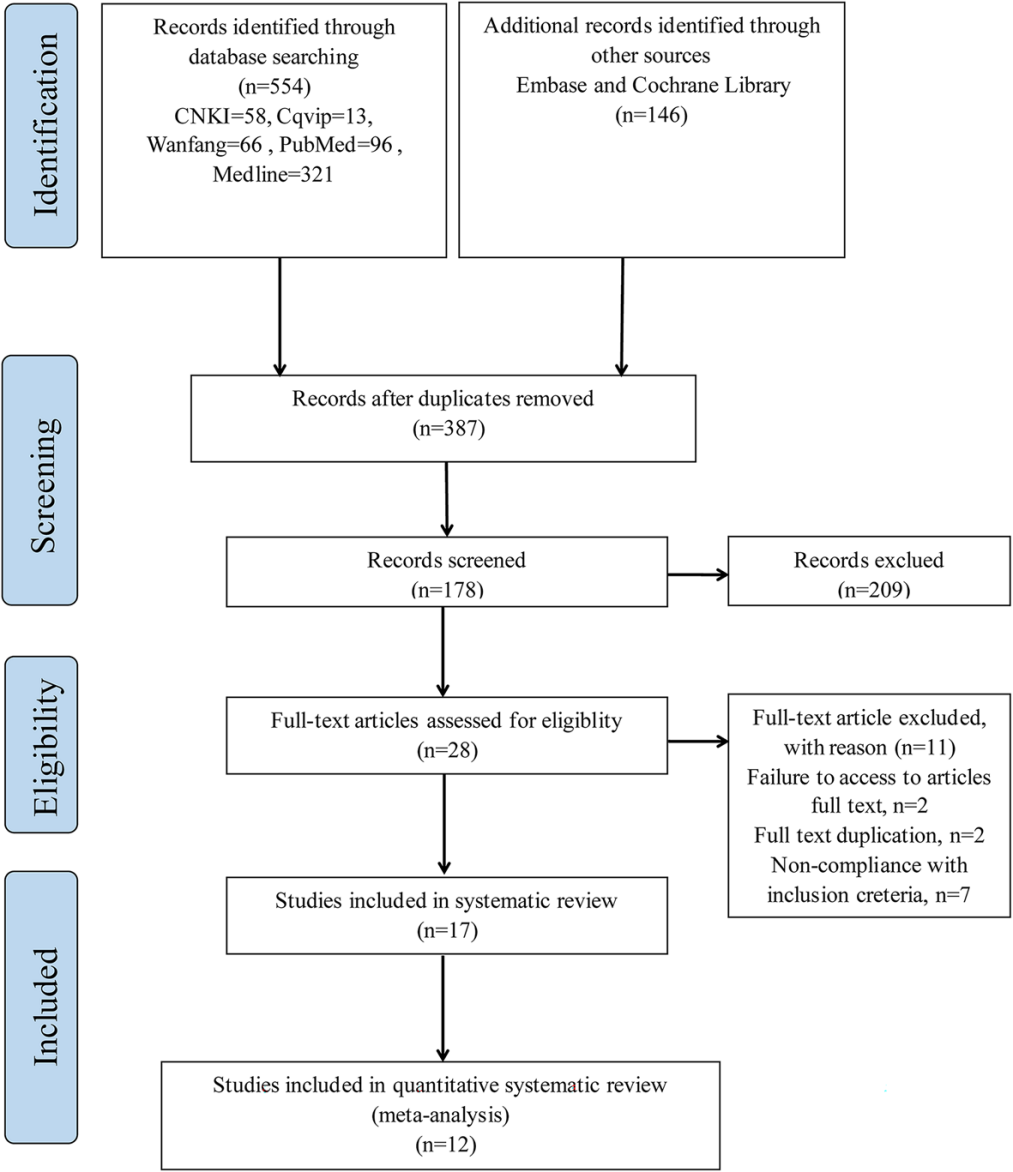
Preferred Reporting Items for Systematic Reviews and Meta-Analyses (PRISMA) flowcharts

The PRISMA flow diagram of the study process is presented in Fig. 1. The search strategy yielded 700 publications (58 from CNKI, 13 from Cqvip, 66 from Wanfang, 96 from PubMed, 96 from Medline, and 146 from other sources), of which 313 were removed as duplicates. After records screening, 209 studies were excluded for not fulfilling the experiment criteria. The full manuscripts of 28 articles were evaluated. In two publications the full text was not accessible, and two of those were excluded for full-text duplication. Seven articles were removed for not meeting the inclusion criteria. Thus, a total of 12 publications with available full texts remained. Finally, we recruited 2548 infertile patients who met the eligibility criteria for quantitative data synthesis in twelve studies: nine RCT studies (*n* = 1773) and three clinical trial studies (*n* = 775) for investigating the effect of vitamin D supplementation on reproductive outcomes. A detailed summary of the included study characteristics is shown in Table 1 and Supplemental Tables S1 and S2.

**Table 1:** main characteristics of the included studies

Author	Year	Country	Study design	Serum Vitamin D concentration before / after supplementary (ng/ml)	Recruited patients number		Treatment(s)	Control	Fertilization	Disease	Duration of Vitamin D supplement
					Case	Control					
Abedi	2019	Iran	Double-Blind Randomized Placebo-Controlled Trial	13.6 ± 6.6/37.1 ± 7.7 vs 12.7 ± 6.4/14.4 ± 6.6	54	54	Vitamin D	placebo	ICSI	Infertile couples who had Vitamin D level below 30 ng/ml without symptom of Vitamin D deficiency	Six weeks
Aflatoonian	2014	Iran	Randomized controlled trial	below 30	57	57	Vitamin D	–	IVF/ICSI	Infertile women undergo IVF/ICSI	Six-eight weeks
Doryanizadeh	2021	Iran	Double-Blind Randomized Clinical Trial	27.5 ± 1.8 vs 27.6 ± 1.8	51	44	Calcitriol (Vitamin D3)	placebo	–	Infertile women	Four weeks
Espinola	2021	Italy	Randomized and controlled pilot study	25.4 (6.7; 16.0–40.0)/33.2(4.3; 23.3–40.4) vs 23.9 (4.9; 14.0–35.6) /24.3(5.2; 16.1–36.4)	60	60	Myo-Inositol (600 mg), folic acid (200 mg), melatonin (1.0 mg) and vitamin D3 (50 μg, 2000 IU) as cholecalciferol	Myo-Inositol (600 mg), folic Acid (200 mg), melatonin (1.0 mg), folic acid (200 μg)	–	Infertile women of different etiology	From the day of hCG administration until 14 days after embryo transfer
Fatemi	2017	Iran	Double-Blind Randomized Placebo-Controlled Trial	below 30	52	53	Vitamin E, 400 mg/day dl alpha tocopherylacetate and vitamin D3	placebo	ICSI	PCOS	Eight weeks
Kermack	2019	United Kingdom	Double-blinded randomized controlled trial	74.33 ± vs 71.62 ± 24.69 nmol/L/ 154.63 ± 1.56 nmol/L vs. 68.50 ± 1.51	55	56	EPA(800 mg), DHA (1200 mg), or vitamin D in olive oil	Sunflower seed oil	IVF or IVF-ICSI	Women undergoing IVF	Six weeks
Lan	2018	China	Clinical trial	below 30	37	37	Vitamin D2	–	–	Infertile women who had failed to undergo IVF fresh embryo transplantation	Six weeks at least
Somigliana	2021	Italy	Randomized superiority double-blind placebo controlled clinical trial	20.0(15.5–23.6) vs 19.9(14.6–23.9)	308	322	Vitamin D3 diluted in olive oil	placebo (the olive oil)	Classical IVF and ICSI	Women undergoing IVF	A single administration
Tang	2017	China	Randomized controlled trial	–	235	155	Multivitamin tablets (elevit)pearl/daily orally	–	IVF-ET	Infertile women	Ninety days
Wdowiak	2020	Poland	Randomized controlled trial	–	50	50	600 mg MI, 200 μg folic acid, 1 mg melatonin, 50 μg equivalent to 2000 IU vitamin D3	placebo	ICSI	Infertile women	Three months
Zhao	2019	China	Clinical trial	–	190	115	25OH-VD	–	–	PCOS and insulin resistance	Two-three months
Zhuang	2019	China	Clinical trial	–	204	192	Vitamin D combined with metformin and clomiphene	metformin and clomiphene	–	Patients with PCOS combined with infertility	Three consecutive menstrual cycles

Author	Administration frequency of Vitamin D	Total Vitamin D dosage (IU)	Administration route	Age (years)	BMI (kg/m2)	Duration of infertility	Transfer type	Stage of embryo	Outcome measures
Abedi	50,000 units/week	300,000	Oral administration	18–38(31.9 ± 4.2/30.8 ± 4.4)	18–30(23.9 ± 2.1/23.8 ± 1.9)	77.4 ± 22.1/68.1 ± 19.3 months	–	–	Biochemical and clinical pregnancy rate
Aflatoonian	50,000/week	300,000-400,000	Oral administration	28.45 ± 3.74/29.56 ± 4.68	26.87 ± 1.77/26.29 ± 1.67	–	Frozen embryo transfer	Embryos A/B/C	Biochemical and clinical pregnancy rate
Doryanizadeh	Two 0.25 μg daily	560	Oral administration	20–40(32.5 ± 4.9/31.6 ± 4.9)	25.3 ± 3.2/24.9 ± 3.4	7.0 ± 4.7/7.1 ± 4.8 years	Frozen embryo transfer	–	Biochemical and clinical pregnancy rate, miscarriage rate and pregnancy continued until week 20
Espinola	50 μg, 2000 IU daily	42,000	Oral administration	≤ 42[34.7 (6.7;22–42)/35.9 (3.7;27.0–42.0)]	18.5–24.9[21.9 (2.1;17.6–28.4)/22.0 (2.3;17.6–27.5)]	3.7 (1.8;1.0–9.0)/3.6 (2.1;1.0–10.0) years	Fresh embryo transfer	Blastocysts graded A/B	Implantation rate, Biochemical and clinical pregnancy rate, miscarriage rate, multiple pregnancy rate
Fatemi	50,000 IU/one in two weeks-3300 IU/daily	200,000	Oral administration	18–38(28.07 ± 4.21/28.13 ± 3.73)	20–34(26.53 ± 2.99/26.13 ± 3.58)	61.61 ± 43.62/66.46 ± 36.31 months	Fresh and frozen embryo transfer	Embryo with good morphologic	Implantation rate, Biochemical and clinical pregnancy rate, multiple pregnancy rate
Kermack	10 μg, 400 IU daily	16,800	Oral administration	18–41(33.3 ± 4.1/33.4 ± 4.3)	18–32(24.3 ± 3.1/25.0 ± 3.9)	–	–	Embryo with highest morphologic score	Implantation, clinical pregnancy and live bith rate
Lan	10 ml (50 mg)/one time in two weeks	45,000	Intramuscular injection	–	–	–	Frozen embryos	–	Implantation and clinical pregnancy rate
Somigliana	600,000 IU	600,000	Oral administration	18–39[35.0(32.0–37.0)/35.0(33.0–37.0)]	18–25[20.8(19.5–22.5)/21.1(19.7–22.9)]	3(2–4)/2.5(2–4) years	Fresh and frozen embryos	Blastocyst Stage (Day 5)	Biochemical and clinical pregnancy rate, miscarriage rate, multiple pregnancy rate and live birth rate
Tang	Vitamin D 500 IU/daily	45,000	Oral administration	24–43(32.5 ± 3.2)/23–42(31.8 ± 3.0)	–	1–12(4.2 ± 1.4)/1–11(4.0 ± 1.2) years	–	–	Clinical pregnancy, and miscarriage rate
Wdowiak	50 μg equivalent to 2000 IU vitamin D3 as cholecalciferol/daily	168,000	Oral administration	20–35(31 ± 3.11/31.2 ± 3.03)	24.76 ± 2.94/25.11 ± 2.39	–	–	–	Clinical pregnancy rate
Zhao	–	–	–	31.2 ± 4.3/32.1 ± 4.2,32.0 ± 3.4/31.6 ± 6.9	22.4 ± 2.4/24.7 ± 4.7, 23.5 ± 3.8/24.1 ± 4.4	3.8 ± 2.3/3.1 ± 3.2,3.7 ± 1.0/3.4 ± 2.2	Frozen embryos	–	Implantation and clinical pregnancy rate
Zhuang	3000 IU daily, 5 days/menstrual cycle	45,000	Oral administration	26.33 ± 4.05/25.64 ± 4.78	27.53 ± 4.13/27.28 ± 3.56	3.87 ± 2.44/3.52 ± 2.56	–	–	Pregnancy rate

### Study characteristics

The main characteristics of the included studies are shown in Table 1. The publication dates of the eligible studies ranged between 2014 and 2021. The number of patients ranged from 74 to 630. Nine studies were RCTs [11–15, 19–21], and three studies were nonrandomized cohort studies [22–24]. The double-blind method was reported in five of the nine RCTs [12, 13, 15, 19, 21]. The risk of bias assessments for the RCTs and cohort studies are summarized in Supplementary Tables S1 and S2. The studies were conducted in Iran (four studies), China (four studies), Italy (two studies), the United Kingdom (one study), and Poland (one study). The serum vitamin D concentration before supplementation was lower than 20 ng/mL in 2 studies, lower than 30 ng/mL in 7 studies, and not limited in 5 studies. The data on serum vitamin D concentration after supplementation were accessible in 3 studies. The patients in the case group underwent vitamin D supplementation in all 12 studies, were treated with vitamin D only in 6 studies, and were multicomponent in 6 studies. The patients in the control group were treated with a placebo in 8 studies and without intervention in 4 studies. The fertilization methods were IVF (one study), IVF/ICSI (three studies), ICSI (three studies), or no information (five studies). All recruited women were infertile and undergoing IVF treatment. Recruited patients with PCOS in three studies or a variety of etiology in seven studies. The duration of vitamin D supplement was in the range of 1–90 days. The administration frequency of vitamin D was daily in 7 studies, weekly in 3 studies, and other 2 in studies. The total vitamin D dosage was in the range of 560–600,000 IU. The administration route of vitamin D was intramuscular injection (one study) or oral administration (ten studies). The embryo transfer type was fresh and frozen embryo transfer (two studies), fresh embryo transfer (one study), frozen embryo transfer (four studies), or undetermined (five studies).

### Effects of Vitamin D supplementation on the reproductive outcomes of infertile patients

**Fig. 2:**
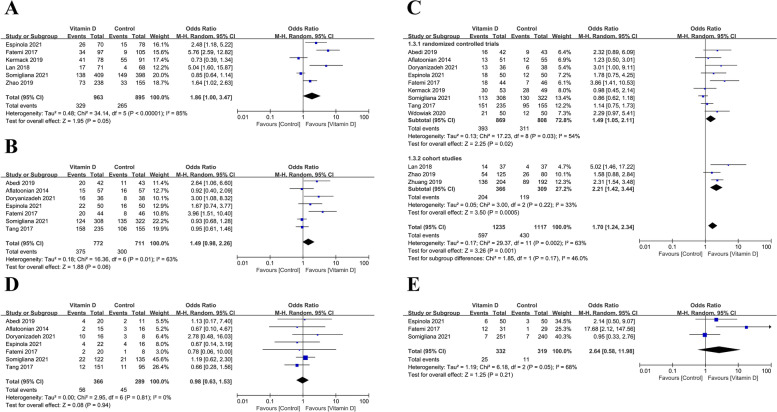
Meta-analyses of the effect of vitamin D supplementation on the reproductive outcomes of infertile patients **A** Implantation; **B** Biochemical pregnancy; **C** Clinical pregnancy; **D** Miscarriage; **E** Multiple pregnancy

The implantation rate outcomes were based on the data derived from 6 studies (963 cases and 895 controls). The implantation rate had no significant difference between the case and control group (OR: 1.86, 95% CI: 1.00–3.47; *P* = 0.05; heterogeneity; *I*^*2*^ = 85%; Fig. 2A).

The biochemical pregnancy rate outcomes were based on the data derived from seven studies (772 cases and 711 controls). The biochemical pregnancy rate had no significant difference in the case group compared with that in the control group (OR: 1.49, 95% CI: 0.98–2.26; *P* = 0.06; heterogeneity; *I*^*2*^ = 63%; Fig. 2B).

The clinical pregnancy rate outcomes were based on the data derived from 12 studies (1235 cases and 1117 controls): nine RCTs and three cohort studies. In RCTs studies, the clinical pregnancy rate was significantly higher in the case group than in the control group (OR: 1.49, 95% CI: 1.05–2.11; *P* = 0.02; heterogeneity; *I*^*2*^ = 54%). In cohort studies, the clinical pregnancy rate was significantly higher in the case group than in the control group (OR: 2.21, 95% CI: 1.42–3.44; *P* = 0.0005; heterogeneity; *I*^*2*^ = 33%). Overall, the clinical pregnancy rate was significantly higher in the case group than in the control group in a total of 11 studies (OR: 1.70, 95% CI: 1.24–2.34; *P* = 0.001; heterogeneity; *I*^*2*^ = 63%; Fig. 2C).

The results of the sensitivity analysis are shown in Supplemental Fig. S1 and S2. It is suggested that data derived from Somigliana (2021) may have a remarkable effect on the merger results (Fig. S2) [13]. Somigliana (2021) was removed, the meta-analysis of the effect of vitamin D supplementation on the clinical pregnancy rate of infertile patients was drawn (Fig. S2) [13]. High heterogeneity suddenly decreased from 63 to 36% (Fig. 2C and S2). The pooled results still indicated that infertile women treated with vitamin D had a significantly increased clinical pregnancy rate compared with the control group (OR: 1.84, 95% CI: 1.39–2.43; *P* < 0.0001; heterogeneity; *I*^2^ = 36%; Fig. S2). And the conclusions of this study were statistically reliable.

However, the miscarriage rate outcomes were based on the data derived from seven studies (366 cases and 289 controls). No difference was found in the miscarriage rate between the case and control group (OR: 0.98, 95% CI: 0.63–1.53; *P* = 0.94; heterogeneity; *I*^*2*^ = 0%; Fig. 2D).

The multiple pregnancy rate outcomes were based on the data derived from three studies (332 cases and 319 controls). The multiple pregnancy rate had no significant difference between the case and control group (OR: 2.64, 95% CI: 0.58–11.98; *P* = 0.21; heterogeneity; *I*^*2*^ = 68%; Fig. 2E).

### Effects of the parameters of vitamin D supplementation on the clinical pregnancy rates of infertile patients

#### The clinical pregnancy rate in studies with different vitamin D levels of infertile patients

**Fig. 3:**
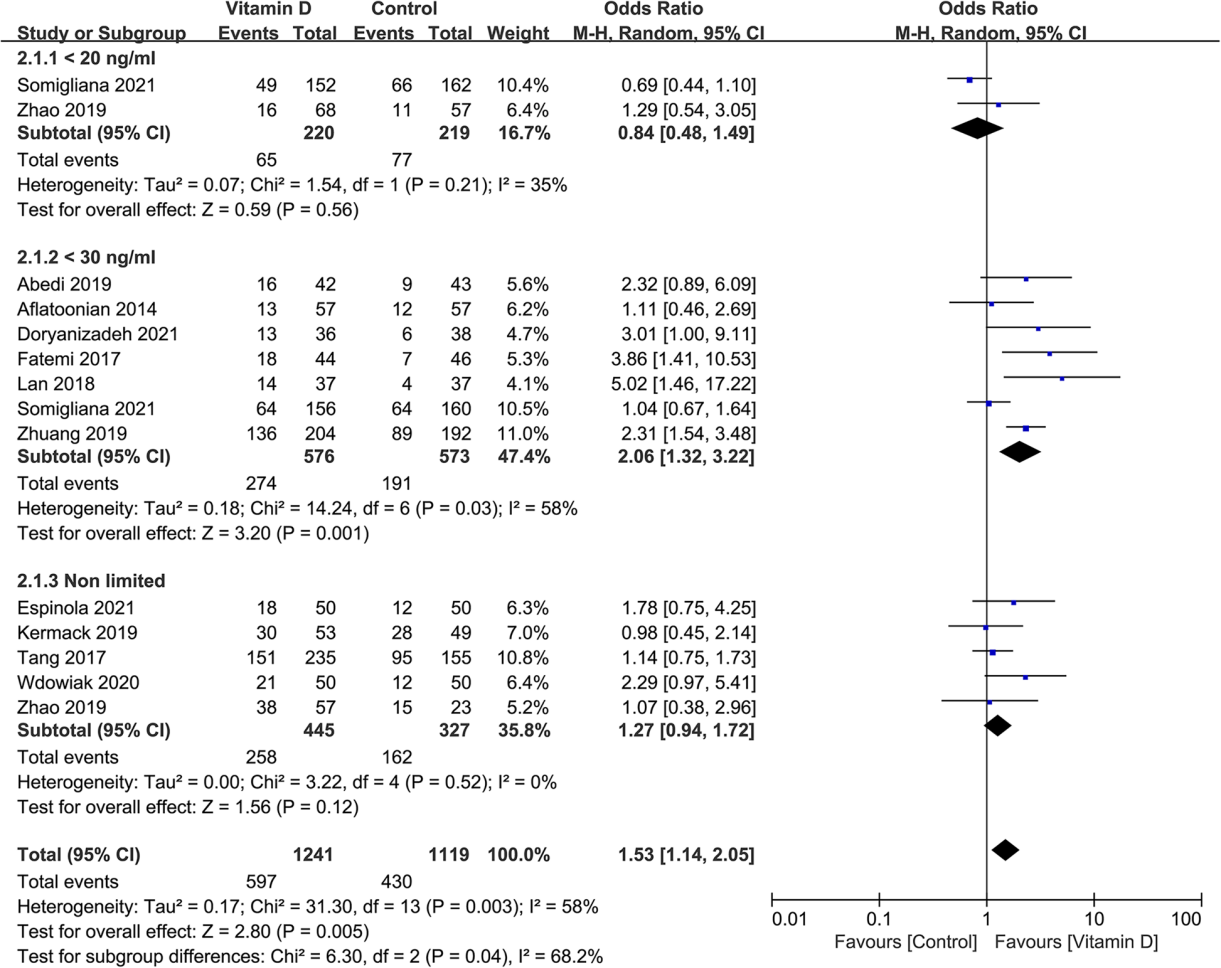
Forrest plot for the effect of vitamin D supplementation on the clinical pregnancy rate in studies with different vitamin D level of infertile patients

No significant difference was found in the clinical pregnancy rate between the case and control groups when the vitamin D level in the serum of infertile patients was lower than 20 ng/mL or had no limited (OR: 0.84, 95% CI: 0.48–1.49; *P* = 0.56; heterogeneity; *I*^*2*^ = 35%; or OR: 1.27, 95%CI: 0.94–1.72; P = 0.12; heterogeneity; *I*^*2*^ = 0%). When the vitamin D level in serum before treatment was lower than 30 ng/mL, the clinical pregnancy rate was significantly increased in the case group than in the control group (OR: 2.06, 95% CI: 1.32–3.22; P = 0.001; heterogeneity; *I*^*2*^ = 58%; Fig. 3).

### The clinical pregnancy rate in studies with different drug types

**Fig. 4:**
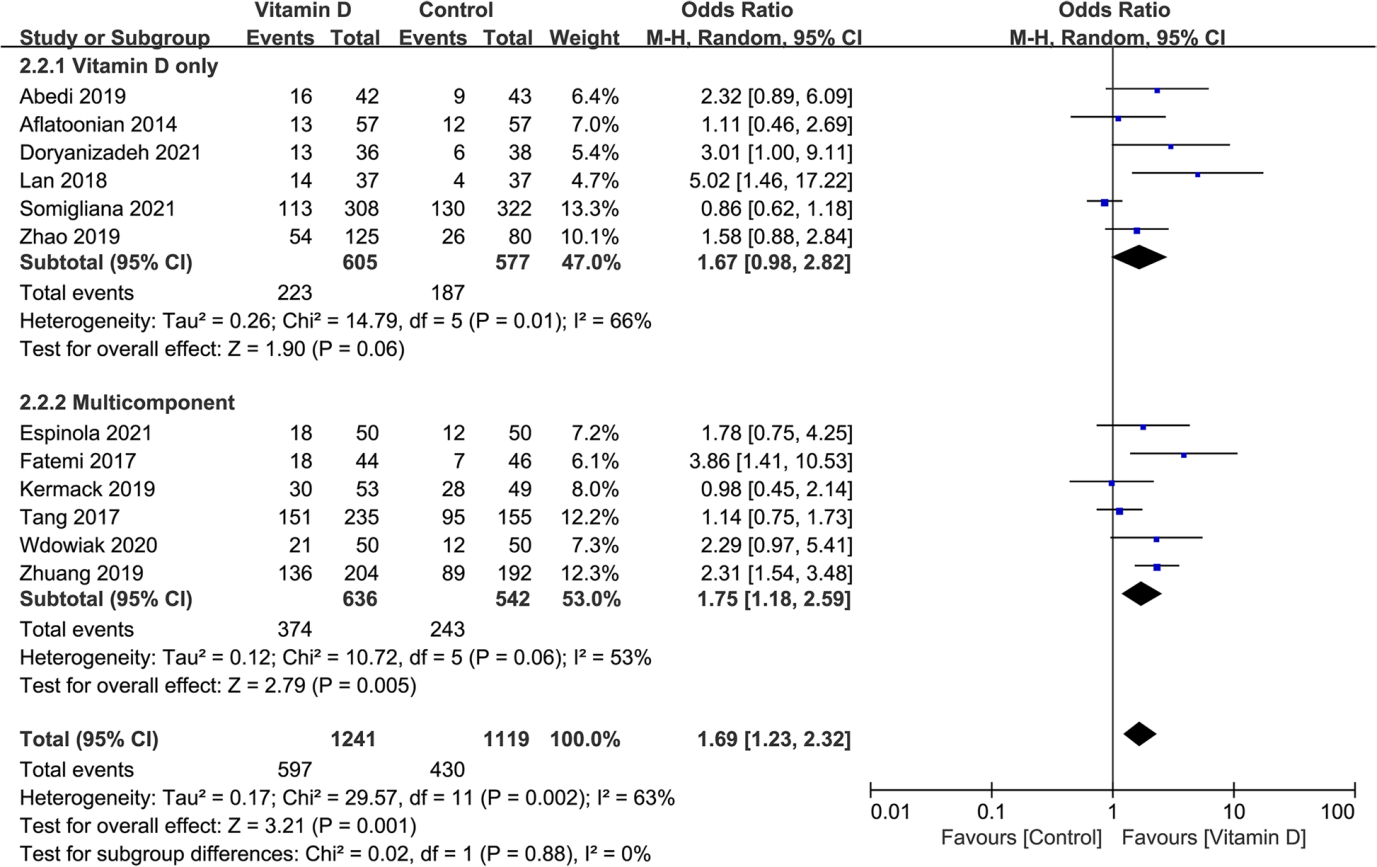
Forrest plot for the effect of vitamin D supplementation on the clinical pregnancy rate in studies with different drug type

When the infertile patients were treated with vitamin D only, the clinical pregnancy rate had no significant difference between the case and control groups (OR: 1.67, 95% CI: 0.98–2.82; *P* = 0.06; heterogeneity; *I*^*2*^ = 66%). However, if the patients got multicomponent drug contained vitamin D, the clinical pregnancy rate was significantly higher in the case group than in the control group (OR: 1.75, 95% CI: 1.18–2.59; *P* = 0.005; heterogeneity; *I*^*2*^ = 53%; Fig. 4).

The results of the sensitivity analysis are shown in Supplemental Fig. S3 and S4. It is suggested that data derived from Somigliana (2021) might have a remarkable effect on the merger results (Fig. S3) [13]. Somigliana (2021) was removed, meta-analysis of the effect of vitamin D supplementation on the clinical pregnancy rate in the subgroup of vitamin D only supplementation was drawn (Fig. S3) [13]. The high heterogeneity suddenly decreased from 66 to 20% (Fig. 4 and S4). The pooled results indicated that infertile women treated with vitamin D only had a significantly increased clinical pregnancy rate compared with the control group (OR: 1.97, 95% CI: 1.26–3.09; *P* < 0.003; heterogeneity; *I*^2^ = 20%; Fig. S4).

### The clinical pregnancy rate in studies with different total dosages of vitamin D supplementation

**Fig. 5:**
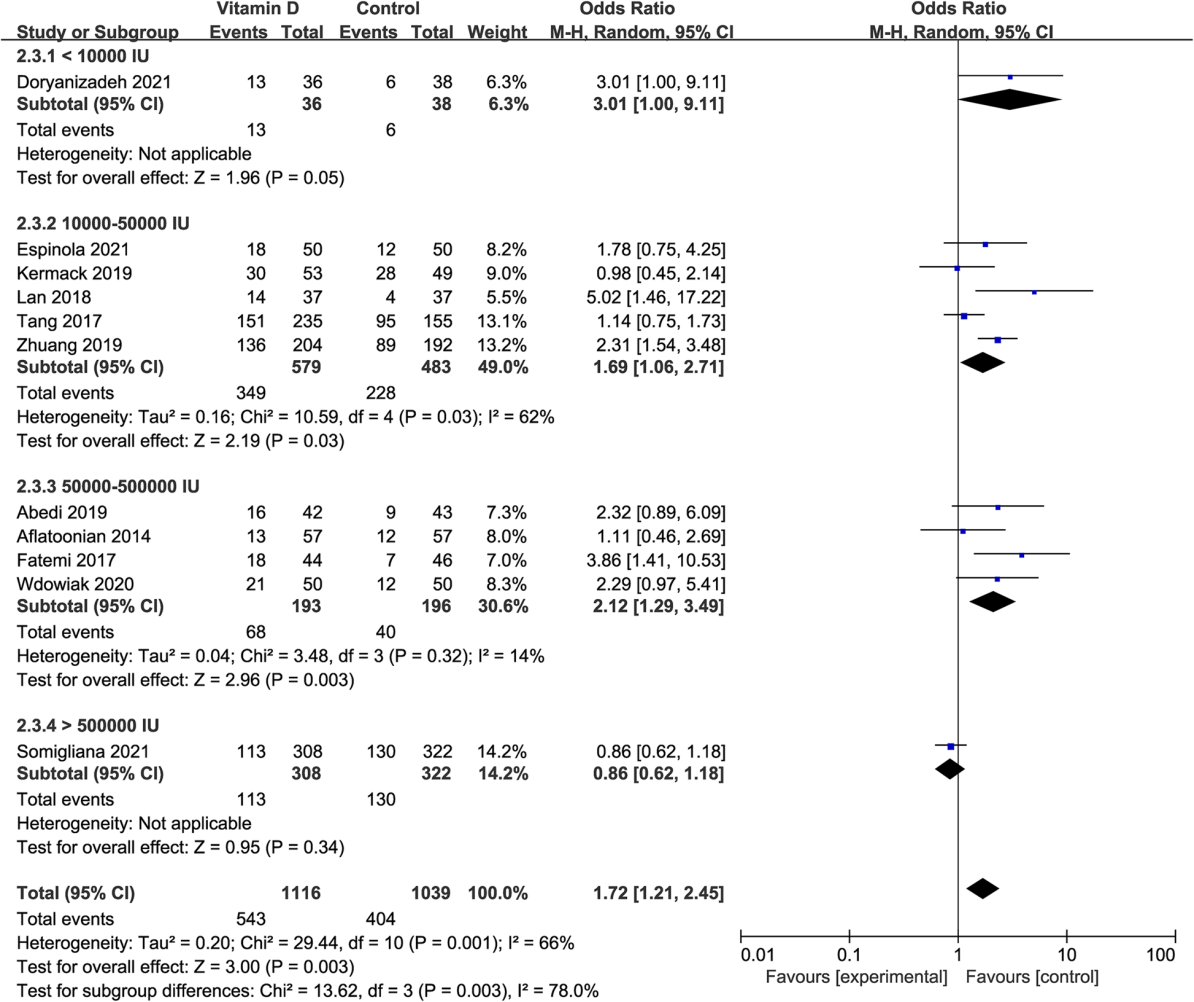
Forrest plot for the effect of vitamin D supplementation on the clinical pregnancy rate in studies with different total dosages of vitamin D supplementation

There was no significant difference in the clinical pregnancy rate between the case and control groups when the total vitamin D dosage was lower than 10,000 IU or higher than 500,000 IU (OR: 3.01, 95% CI: 1.00–9.11; *P* = 0.05; or OR: 0.86, 95% CI: 0.62–1.18; *P* = 0.34). Compared with the control group, the clinical pregnancy rate increased significantly in the case group when the infertile patients were treated with 10,000–50,000 IU or 50,000–500,000 IU vitamin D during the whole supplementation (OR: 1.69, 95% CI: 1.06–2.71; *P* = 0.03; heterogeneity; *I*^*2*^ = 62%; or OR: 2.12, 95% CI: 1.29–3.49; *P* = 0.003; heterogeneity; *I*^*2*^ = 14%; Fig. 5).

### The clinical pregnancy rate in studies with different duration of vitamin D supplementation

**Fig. 6:**
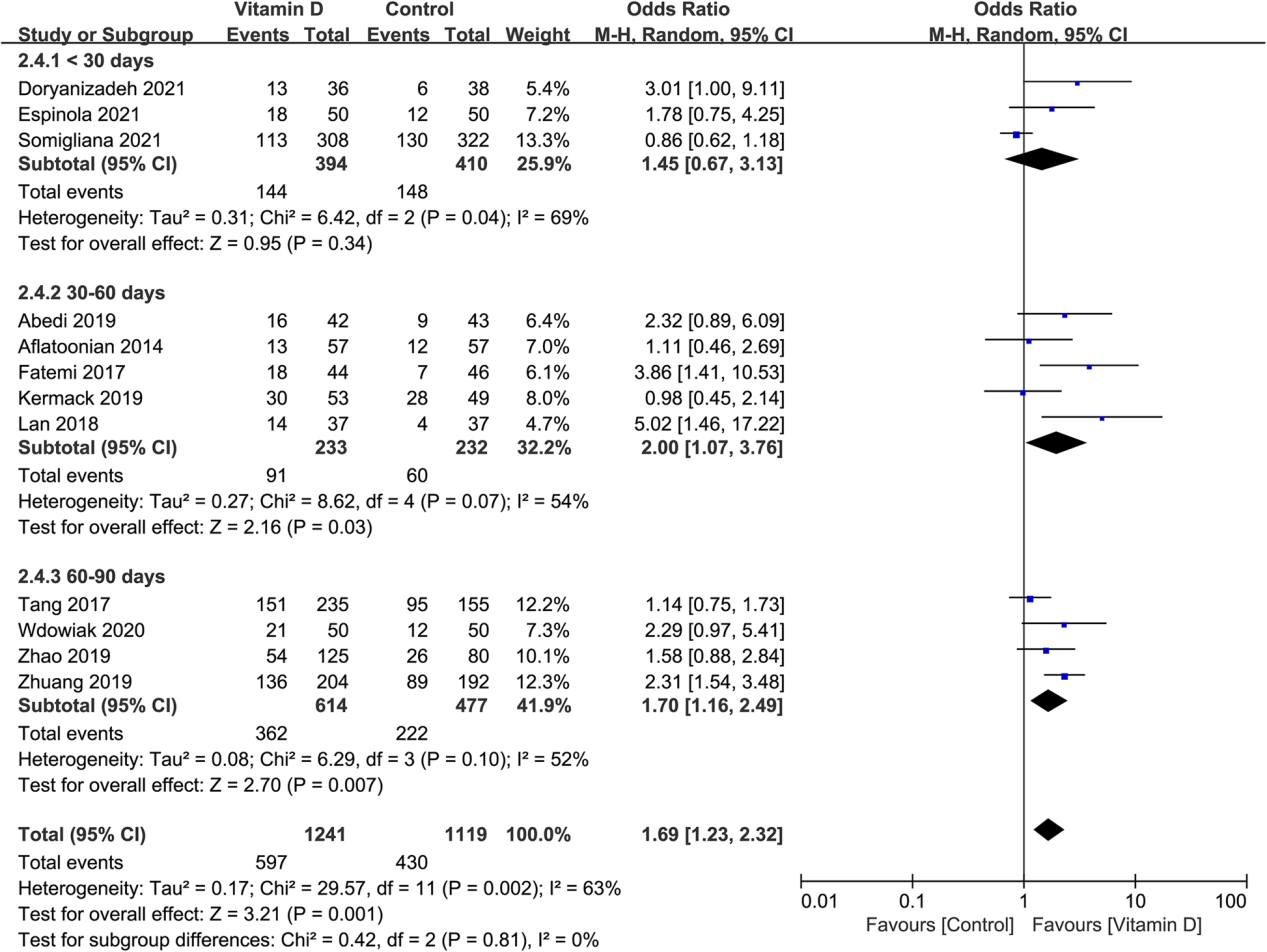
Forrest plot for the effect of vitamin D supplementation on the clinical pregnancy rate in studies with different duration of vitamin D supplementation

The clinical pregnancy rate was similar in the case group compared with the control group when the duration of vitamin D supplementation was shorter than 30 days (OR: 1.45, 95% CI: 0.67–3.13; *P* = 0.34; heterogeneity; *I*^*2*^ = 69%). When the vitamin D supplementation lasted for 30–60 days or 60–90 days, the clinical pregnancy rate was significantly higher in the case group than in the control group (OR: 2.00, 95% CI: 1.07–3.76; *P* = 0.03; heterogeneity; *I*^*2*^ = 54%; or OR: 1.70, 95% CI: 1. 16–2.49; *P* = 0.007; heterogeneity; *I*^*2*^ = 52%; Fig. 6).

### The clinical pregnancy rate in studies with different administration frequencies of vitamin D supplementation

**Fig. 7:**
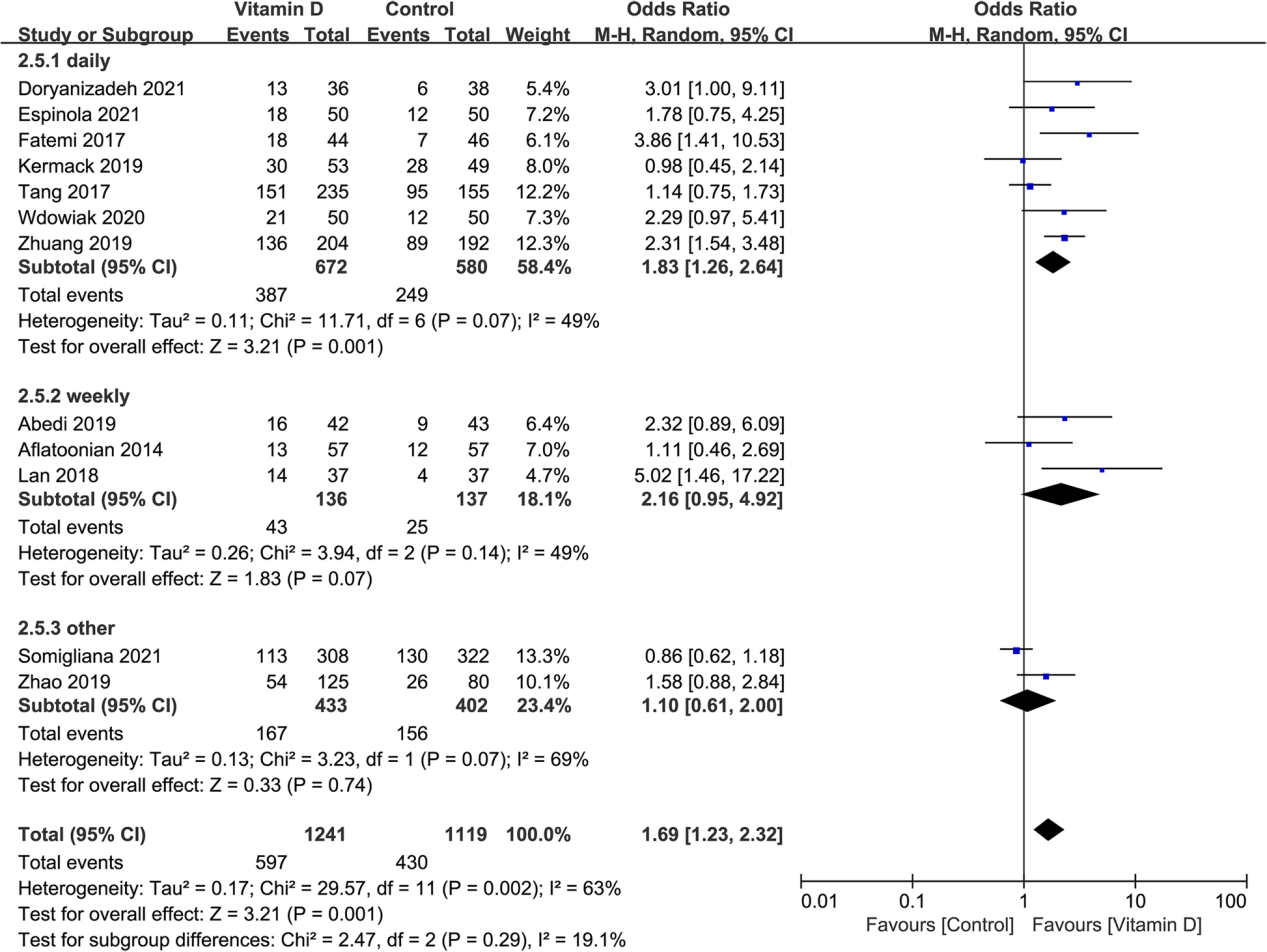
Forrest plot for the effect of vitamin D supplementation on the clinical pregnancy rate in studies with different administration frequency of vitamin D supplementation

The clinical pregnancy rate was significantly higher in the case group compared with the control group when vitamin D supplementation was given every day or weekly (OR: 1.83, 95% CI: 1.26–2.64; *P* = 0.001; heterogeneity; *I*^*2*^ = 49%; or OR: 2.16, 95% CI: 0.95–4.92; *P* = 0.07; heterogeneity; *I*^*2*^ = 49%). When the vitamin D was administrated at one time or other frequency, the clinical pregnancy rate was similar in the case group compared with the control group (OR: 1.10, 95% CI: 0.61–2.00; *P* = 0.74; heterogeneity; *I*^*2*^ = 69%; Fig. 7).

### The clinical pregnancy rate in studies with different dosages of vitamin D supplementation daily

**Fig. 8:**
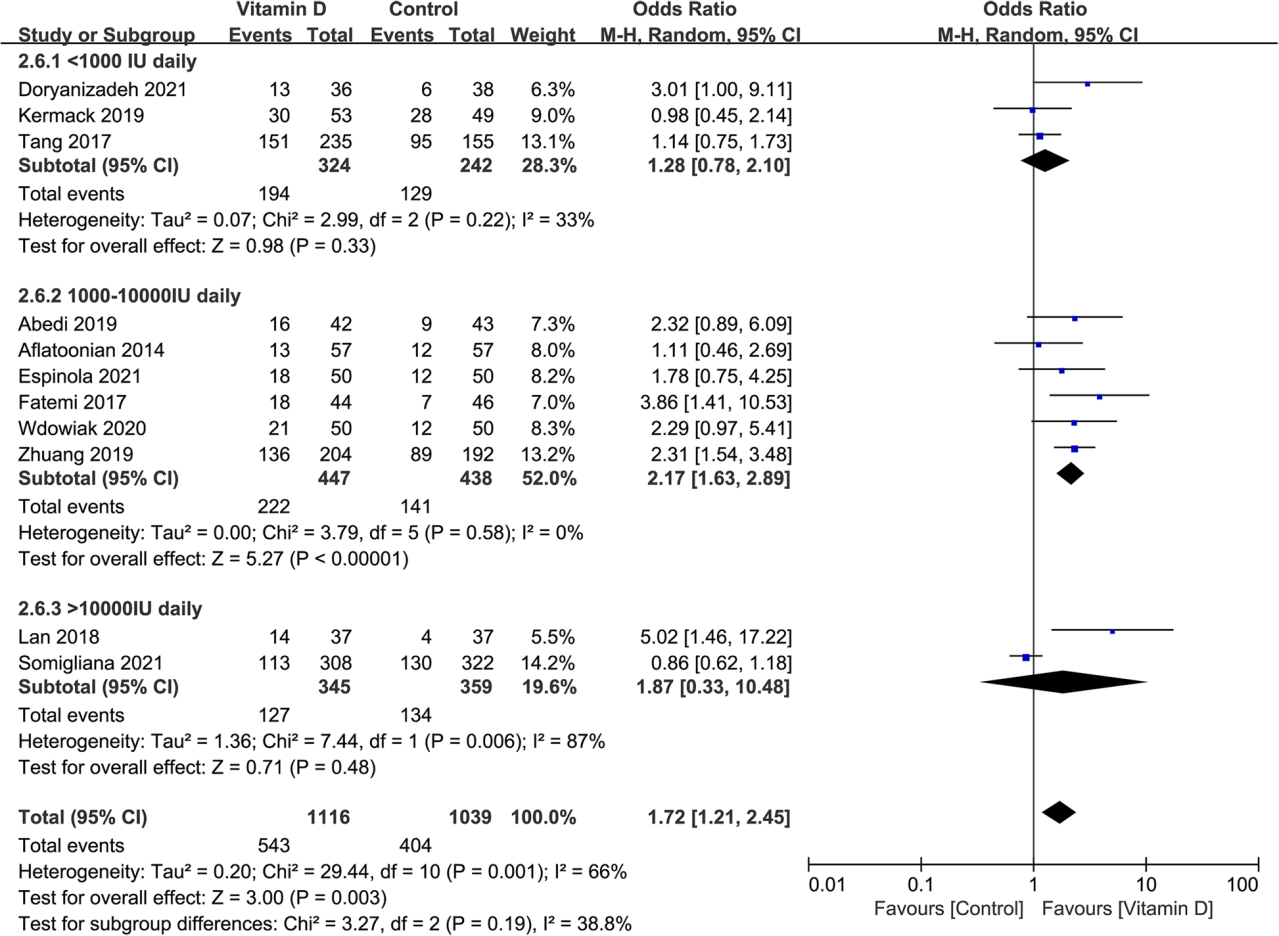
Forrest plot for the effect of vitamin D supplementation on the clinical pregnancy rate in studies with different dosage of vitamin D supplementation daily

The clinical pregnancy rate was similar in the case group compared with the control group when the dosage of vitamin D supplementation daily was lower than 1000 IU (OR: 1.28, 95% CI: 0.78–2.10; *P* = 0.33; heterogeneity; *I*^*2*^ = 33%). When the dosage of vitamin D supplementation daily ranged from 1000 to 10,000 IU, the clinical pregnancy rate was significantly higher in the case group than in the control group (OR: 2.17, 95% CI: 1.63–2.89; *P* < 0.00001; heterogeneity; *I*^*2*^ = 0%). Compared with the control group, the clinical pregnancy rate was the same in the case group when the dosage of vitamin D supplementation daily was higher than 10,000 IU (OR: 1.87, 95% CI: 0.33–10.48; *P* = 0.48; heterogeneity; *I*^*2*^ = 87%; Fig. 8).

## Discussion

This study demonstrated that vitamin D supplementation successfully improved the clinical pregnancy rate of infertile women, but failed to significantly alter the implantation and biochemical pregnancy rate. However, we found that the results were significantly influenced by the article reported by Somigliana et al. [13]. When the data from this article was removed, the implantation and biochemical pregnancy rate significantly increased [13]. The alteration might be caused by its research design [13]. Somigliana et al. designed that the patients took a single oral dose of 600,000 IU [13]. This single dosage was much higher than the maximum dose of supplementation for vitamin D-deficient adults recommended by the Scientific Advisory Committee on Nutrition (SACN) which should not exceed 4000 IU/day or suggested by the National Institute for Health and Care Excellence (NICE) 50,000 IU per week for 6 weeks (300,000 IU in total) [25, 26]. Even though vitamin D supplementation was suggested as a safe and well-tolerated intervention, the drug dosage of clinical intervention still needed careful consideration [16, 27–29]. Especially, the previous reports proposed that there were toxicity and counterproductive influence when serum vitamin D concentrations greater than 150 ng/mL (greater than 374 nmol/L) [16, 27, 28, 30]. Even previous articles showed that large bolus vitamin D dose could be cleared within a week, achieving little or no detectable effect on circulating the vitamin D status [31, 32]. All of these reasons could be used to explain the results bias caused by Somigliana et al. [13]. Increased clinical pregnancy rate might be associated with successful implantation, not resulting from reducing the risk of miscarriage. The results supported the hypothesis that vitamin D exerted pivotal effects on initial embryo implantation, the early trophoblast invasion, and the decidualization of endometrium, not on the second-trimester loss for infertile women undergoing IVF treatment [33, 34].

Many previous reports proposed that the low level of vitamin D was related to poor implantation and infertility [9, 35]. The cut-off value of serum vitamin D was adopted by the Endocrine Society [36]. The serum 25-hydroxy vitamin D_3_ concentration of <20 ng/mL was considered vitamin D deficiency, 21–29 ng/mL was considered insufficient, and > 30 ng/mL was considered replete [36]. We separated the recruited population according to these vitamin D levels into three groups and tried to check whether the vitamin D level before the supplementation could affect the reproductive outcomes of the vitamin D treatment. Only the patients whose vitamin D level was lower than 30 ng/mL could benefit from the supplementation, neither the vitamin D concentration in serum lower than 20 ng/mL nor non-limited. These results could be explained by the hypothesis that individuals with different genotypes of vitamin D-related genes had different responses to vitamin D supplementation [37]. Polymorphism in several vitamin D genes (CYP2R1, CYP27A1, CYP27B1, CYP24A1, VDBP, and VDR) had been associated with vitamin D metabolism and regulated the activity of vitamin D [37]. Single nucleotide polymorphisms (SNP) in GC (rs4588 and rs7041), VDR (rs10735810), and CYP27B1 (rs10877012) also were reported associated with vitamin D status [38, 39]. GC (rs4588 and rs2282679) were associated with lower vitamin D status both before and after vitamin D supplementation [37]. So the patients with vitamin D status lower than 20 ng/mL might carry related genes with poor vitamin D response, no significant benefit was provided. Overall, vitamin D supplementation was encouraged for infertile patients with vitamin D status lower than 30 ng/mL.

The previous article showed that a short period of dietary intervention containing omega-3 Fas and vitamin D could improve the quality of embryo cleavage [21]. Our results showed that the individual components (vitamin D only) resulting in improved clinical pregnancy rate might be underdetermined. The multicomponent including Myo-Inositol, folic acid, melatonin vitamin E and D ect, improved the pregnancy rate which confirmed that not vitamin D exerted a positive influence on reproductive outcomes independently but synergistically. However, the sensitivity analysis (the exclusion of the study by Somigliana et al.) showed that infertile women treated with vitamin D only also had a significantly increased clinical pregnancy rate compared with the control group [13]. More researches about the effect of vitamin D supplementation on the clinical pregnancy rate with different drug type were needed.

Vitamin D is a fat-soluble steroid hormone, has lipophilic nature, and distributes in adipose tissue [40, 41]. Vitamin D has a slow turnover in the body with a half-life of approximately 2 months [40, 42]. Vitamin D could be metabolized by 25-hydroxylase, a liver enzyme, into 25(OH) D which has a half-life of 15 days [40, 42]. The (25(OH)D) again could be converted into calcitriol or 1,25(OH)_2_ D by enzyme CYP27B1 [40, 42]. 1,25(OH)_2_ D has a half-life of 15 hours [40, 43]. The pharmacokinetics of vitamin D can impact the effects of vitamin D supplementation, so the dosing regimen of vitamin D supplementation had to be taken into consideration. To maximize the chance of achieving pregnancy and minimize and minimize the detrimental and toxicity effects of vitamin D supplementation, we set the subgroup of total vitamin D dosage, duration, administration frequency, and daily vitamin D dosage to confirm the suitable intervention. When the total vitamin D dosage was too low (lower than 10,000 IU) or too high (higher than 500,000 IU), the clinical pregnancy rate had no significant increase. The total vitamin D dosage ranged from 10,000–500,000 IU might be proper for infertile patients. The infertile patients could achieve better reproductive outcomes when they got vitamin D (1,000–10,000 IU) supplementation every day that lasts for more than 30 days. In comparison to the vitamin D administrated weekly or at others interverals (monthly or longer intervals), this study yielded only positive results for daily treatment. That could be explained by the hypothesis that only daily vitamin D supplementation could maintain stable circulating concentrations over time [31, 44]. The infertile patients treated with vitamin D dose varied from 1000-10,000 IU daily could benefit from the supplementation. A dose lower than 1000 IU or higher than 10,000 IU daily failed to show that vitamin D could improve the clinical pregnancy rate of infertile patients. These results indicated that patients treated with a small daily dose might still be at risk of vitamin D deficiency, so the improvement had failed. This finding was consistent with the past researches that approximately 280 IU/d or 400 IU/d dose for several months had minimal, or even no effect on the circulating vitamin D [44, 45]. While large bolus dosing with vitamin D caused a dramatic fluctuation circulating 25(OH) D levels, which have little benefit, or even be adverse [46, 47]. That might be because the sudden increased vitamin D levels caused by the bolus vitamin D could trigger countervailing factors. Low response to bolus dosing of vitamin D leaded to increase of vitamin D level not as expected [48, 49]. 24-hydroxylase (CYP24A1) up-regulated by the bolus dosing of vitamin D could significantly increase 24,25(OH)_2_D_3_, down-regulate 1,25(OH)_2_D and inhibits immune-modulation for weeks or even months [48–50]. We summarized and discussed that moderate daily dosing of vitamin D supplementation was an appropriate dosing regimen. A suitable vitamin D dosing regimen could have positive effects on the clinical pregnancy rate of infertile patients.

Even though several clinical parameters were analyzed to figure out which parameter might regulate the reproductive outcomes, several limitations still existed in our study. The limitations mainly originated from the clinical heterogeneity of the included publications, including the different ethnicities, uncertain vitamin D status before and after vitamin D supplementation, duration of vitamin D supplementation, and the recruited infertile women of different etiology. Even though vitamin D supplementation was thought a safe and low-cost treatment, we still found the variation of vitamin D supplementation was quite large. Proper doses of vitamin D supplementation should be determined. Furthermore, infertile women in 3 articles had been shown that their serum vitamin D level got significantly increased after the intervention. The lack of vitamin D data after the intervention might mean it was possible vitamin D insufficient or deficiency was not changed, and the full effect of the intervention was not elicited. It is necessary to monitor the response to vitamin D supplements. The analysis of subgroups, according to the duration of vitamin D supplementation, should not be overlooked. The heterogeneity was high in all subgroups, so the result might be not reliable. This might possible because the parameter - duration was not an independent factor influencing the clinical pregnancy rate. The duration of vitamin D supplementation could be affected by the administration frequencies and total dosages of vitamin D supplementation. Patients with different genotypes have different responses to the supplementation, so how the guide medication according to the genotype also should be paid attention to. Vitamin D could be self-synthesized by the human body, and the level of vitamin D is vitiated with the seasons’ change. Whether the vitamin D supplementation should be adjusted according to the seasons is to be considered in the future. Recognizing the limitations of studies included in meta-analyses may stimulate future studies with better designs and methods that will improve available evidence and definitively define the role of vitamin D in ART.

## Conclusion

Our study provides important evidence to support that taking appropriate vitamin D in combination with other components, before pregnancy, can increase reproductive outcomes, but not prevent infertile women from experiencing miscarriages. What’s more, women taking vitamin D supplements can be affected by the parameters of vitamin D. And the infertile patients at risk of vitamin D deficiency received moderate daily dosing of vitamin D supplementation are more likely to have good reproductive outcomes. However, the included articles have a small sample size and high heterogeneity, so further investigating the mechanism of vitamin D treatment acting on the infertile population is still necessary.

## Supplementary Information

**Additional files 1: Table S1.** Risk of bias assessment of the randomized controlled trials for meta-analysis using the Cochrane tool. **Table S2**. Quality assessment of the cohort studies for the meta-analysis using the Newcastle-Ottawa scale.

**Additional files 2: Fig. S1.** Sensitivity analysis for the effect of vitamin D supplementation on clinical pregnancy rate of infertile patients using random effect model (Odds Ratio).

**Additional files 3: Fig. S2.** Forrest plot for the effect of vitamin D supplementation on the clinical pregnancy rate of infertile patients [leave Somigliana (2021) out].

**Additional files 4: Fig. S3.** Sensitivity analysis for the effect of vitamin D supplementation on the clinical pregnancy rate in studies of vitamin D only supplementation using random effect model (Odds Ratio).

**Additional files 5: Fig. S4.** Forrest plot for the effect of vitamin D supplementation on the clinical pregnancy rate in studies of vitamin D only supplementation [leave Somigliana (2021) out].

## Data Availability

The datasets used and/or analyzed during this study are available in this published article and supplementary.
