# Supplementary material for: Influence of Vitamin D supplementation on reproductive outcomes of infertile patients: a systematic review and meta-analysis
Source: Reprod Biol Endocrinol. 2023 Feb 3;21:17. doi: 10.1186/s12958-023-01068-8 (PMC9896710; doi:10.1186/s12958-023-01068-8)
Supplement: Supplementary file 1 — Additional files 1: Table S1. Risk of bias assessment of the randomized controlled trials for meta-analysis using the Cochrane tool. Table S2. Quality assessment of the cohort studies for the meta-analysis using the Newcastle-Ottawa scale. [file 12958_2023_1068_MOESM1_ESM.docx]

Search strategy

1. ART/Assisted Reproductive Technique/Reproductive Technique, Assisted/Technique, Assisted Reproductive/Techniques, Assisted Reproductive/Assisted Reproductive Technics/Assisted Reproductive Technic/Reproductive Technic, Assisted/Reproductive Technics, Assisted/Technic, Assisted Reproductive/Technics, Assisted Reproductive/Assisted Reproductive Techniques/Reproductive Technology, Assisted/Assisted Reproductive Technologies/Assisted Reproductive Technology/Reproductive Technologies, Assisted/Technologies, Assisted Reproductive/Technology, Assisted Reproductive
2. ET/Embryo Transfers/Transfer, Embryo/Transfers, Embryo/Blastocyst Transfer/Tubal Embryo Transfer/Tubal Embryo Stage Transfer
3. ICSI/Injection, Intracytoplasmic Sperm/Injections, Intracytoplasmic Sperm/Intracytoplasmic Sperm Injection/Sperm Injection, Intracytoplasmic/Intracytoplasmic Sperm Injections/Injections, Sperm, Intracytoplasmic
4. IVF/In Vitro Fertilization/In Vitro Fertilizations/Test-Tube Fertilization/Fertilization, Test-Tube/Fertilizations, Test-Tube/Test Tube Fertilization/Test-Tube Fertilizations/Fertilizations in Vitro/Test-Tube Babies/Babies, Test-Tube/Baby, Test-Tube/Test Tube Babies/Test-Tube Baby
5. 1 OR 2 OR 3 OR 4
6. Vitamin D/25 hydroxyvitamin D /1,25 dihydroxyvitamin D3/cholecalciferol
7. 1,25-(OH)2D3/1,25-Dihydroxycholecalciferol/1,25 Dihydroxycholecalciferol/1,25-Dihydroxyvitamin D3/1,25 Dihydroxyvitamin D3/D3, 1,25-Dihydroxyvitamin/1 alpha,25-Dihydroxycholecalciferol/1 alpha,25-Dihydroxyvitamin D3/1 alpha,25 Dihydroxyvitamin D3/D3, 1 alpha,25-Dihydroxyvitamin/Bocatriol/Calcijex/Calcitriol KyraMed/KyraMed, Calcitriol/Calcitriol-Nefro/Calcitriol Nefro/Decostriol/MC1288/MC-1288/MC 1288/Osteotriol/Renatriol/Rocaltrol/Silkis/Sitriol/Soltriol/Tirocal/20-epi-1alpha,25-dihydroxycholecaliferol/1,25-dihydroxy-20-epi-Vitamin D3/1,25 dihydroxy 20 epi Vitamin D3/D3, 1,25-dihydroxy-20-epi-Vitamin/1,25(OH)2-20epi-D3/1 alpha, 25-dihydroxy-20-epi-Vitamin D3
8. Calciol/(3 beta,5Z,7E)-9,10-Secocholesta-5,7,10(19)-trien-3-ol/Vitamin D 3/Vitamin D3/Cholecalciferols
9. ergocalciferol OR alphacalcidol OR alfacalcidol OR calcitriol OR paricalcitol OR doxerocalciferol
10. 6 OR7 OR 8 OR 9 OR 9
11. 5 AND 10

Table S1 Risk of bias assessment of the randomized controlled trials for meta-analysis using the Cochrane tool

| Study | Selection bias | | Performance bias | Detection bias | Attrition bias | Reporting bias |
| --- | --- | --- | --- | --- | --- | --- |
|  | Random sequence generation | Allocation concealment | Blinding of participants and personnel | Blinding of outcome | Incomplete outcome data | Selective reporting |
| Abedi (2019) | Low | Low | Low | Low | Low | Low |
| Aflatoonian (2014) | Low | Low | High | High | Low | Low |
| Doryanizadeh (2021) | Low | Low | Unclear | Unclear | Low | Low |
| Espinola (2021) | Low | Low | High | High | Low | Low |
| Fatemi (2017) | Low | Low | Low | Low | Low | Low |
| Kermack (2019) | Low | Low | Low | Low | Low | Low |
| Somigliana (2021) | Low | Low | Low | Low | Low | Low |
| Tang (2017) | Low | Low | High | High | Unclear | Unclear |
| Wdowiak (2020) | Low | Low | High | High | Unclear | Unclear |

Table S2 Quality assessment of the cohort studies for the meta-analysis using the Newcastle-Ottawa scale

| Study | Selection | | | | Comparability | Exposure | | | Score |
| --- | --- | --- | --- | --- | --- | --- | --- | --- | --- |
|  | Definition of cases | Representativeness of cases | Selection of controls | Definition of controls | Comparability of cases and controls on the basis of the design or analysis | Ascertainment of exposure | Same method of ascertainment for cases and controls | Nonresponse rate |  |
| Lan (2018) | 1 | 1 | 0 | 1 | 2 | 0 | 0 | 0 | 5 |
| Zhao (2019) | 1 | 1 | 0 | 1 | 2 | 1 | 1 | 0 | 7 |
| Zhuang (2019) | 1 | 1 | 0 | 1 | 2 | 1 | 1 | 0 | 7 |
