# Supplementary figures and images for: Influence of Vitamin D supplementation on reproductive outcomes of infertile patients: a systematic review and meta-analysis
Source: Reprod Biol Endocrinol. 2023 Feb 3;21:17. doi: 10.1186/s12958-023-01068-8 (PMC9896710; doi:10.1186/s12958-023-01068-8)

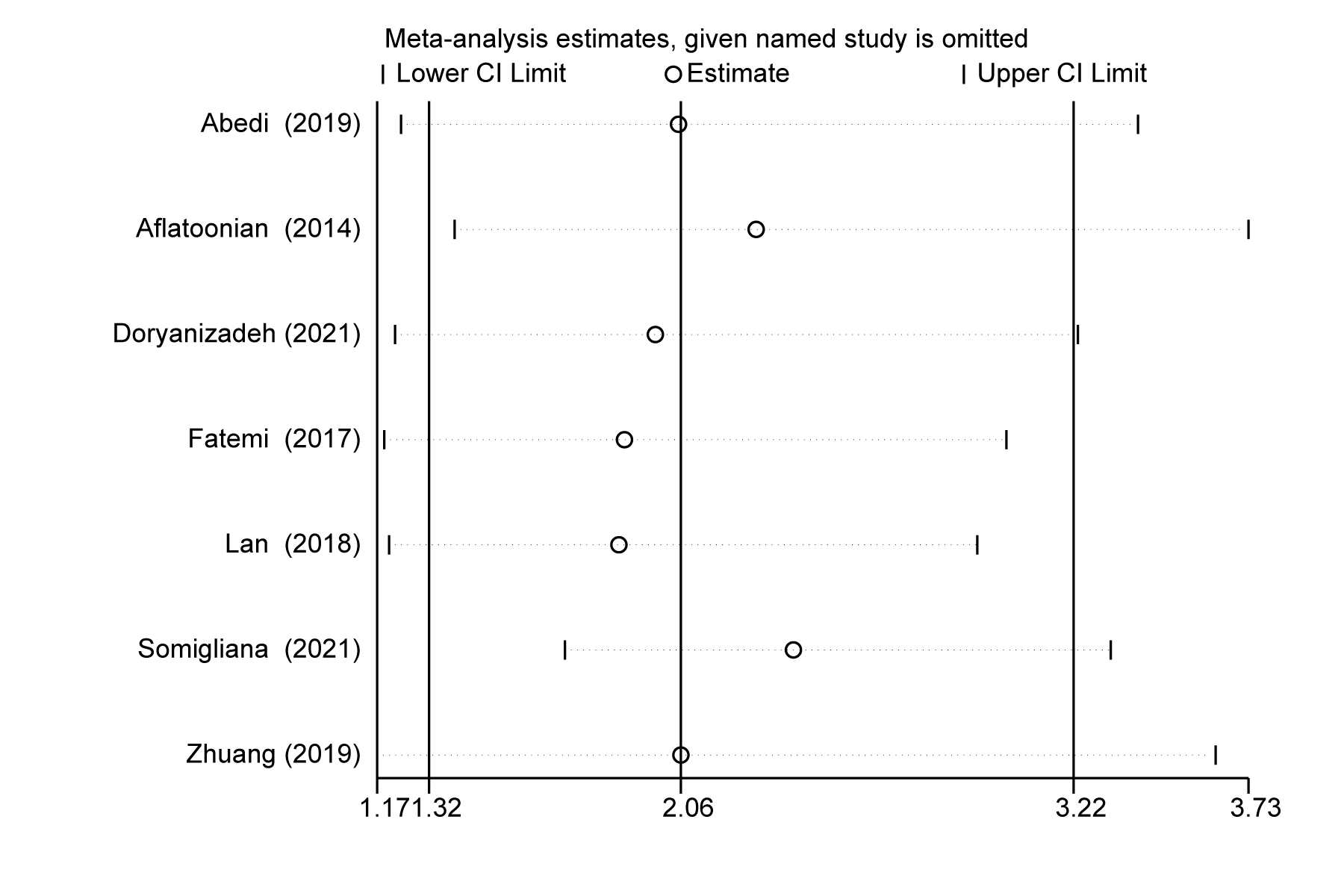

Supplement: Supplementary file 2 — Additional files 2: Fig. S1. Sensitivity analysis for the effect of vitamin D supplementation on clinical pregnancy rate of infertile patients using random effect model (Odds Ratio). [file 12958_2023_1068_MOESM2_ESM.tif]

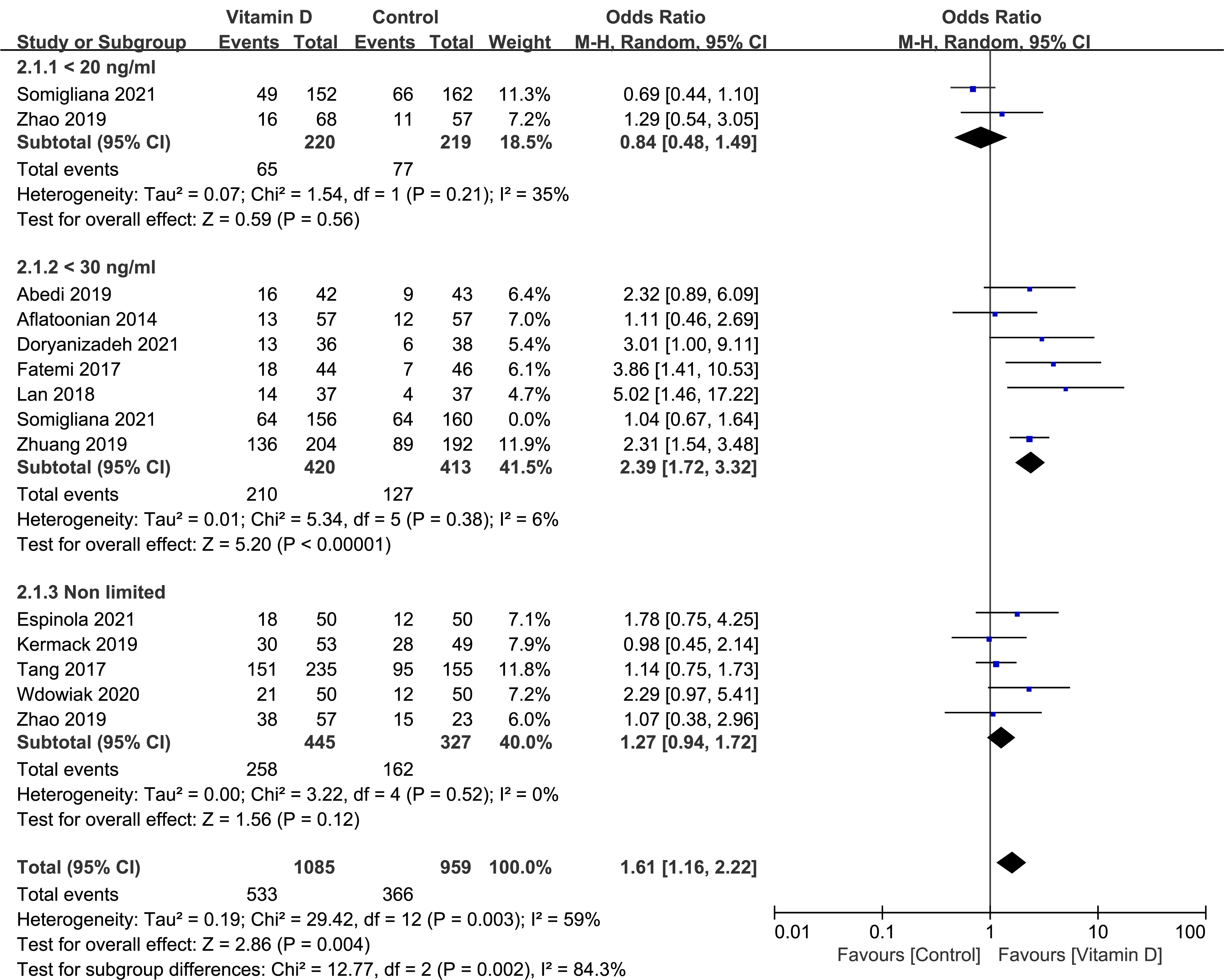

Supplement: Supplementary file 3 — Additional files 3: Fig. S2. Forrest plot for the effect of vitamin D supplementation on the clinical pregnancy rate of infertile patients [leave Somigliana (2021) out]. [file 12958_2023_1068_MOESM3_ESM.tif]

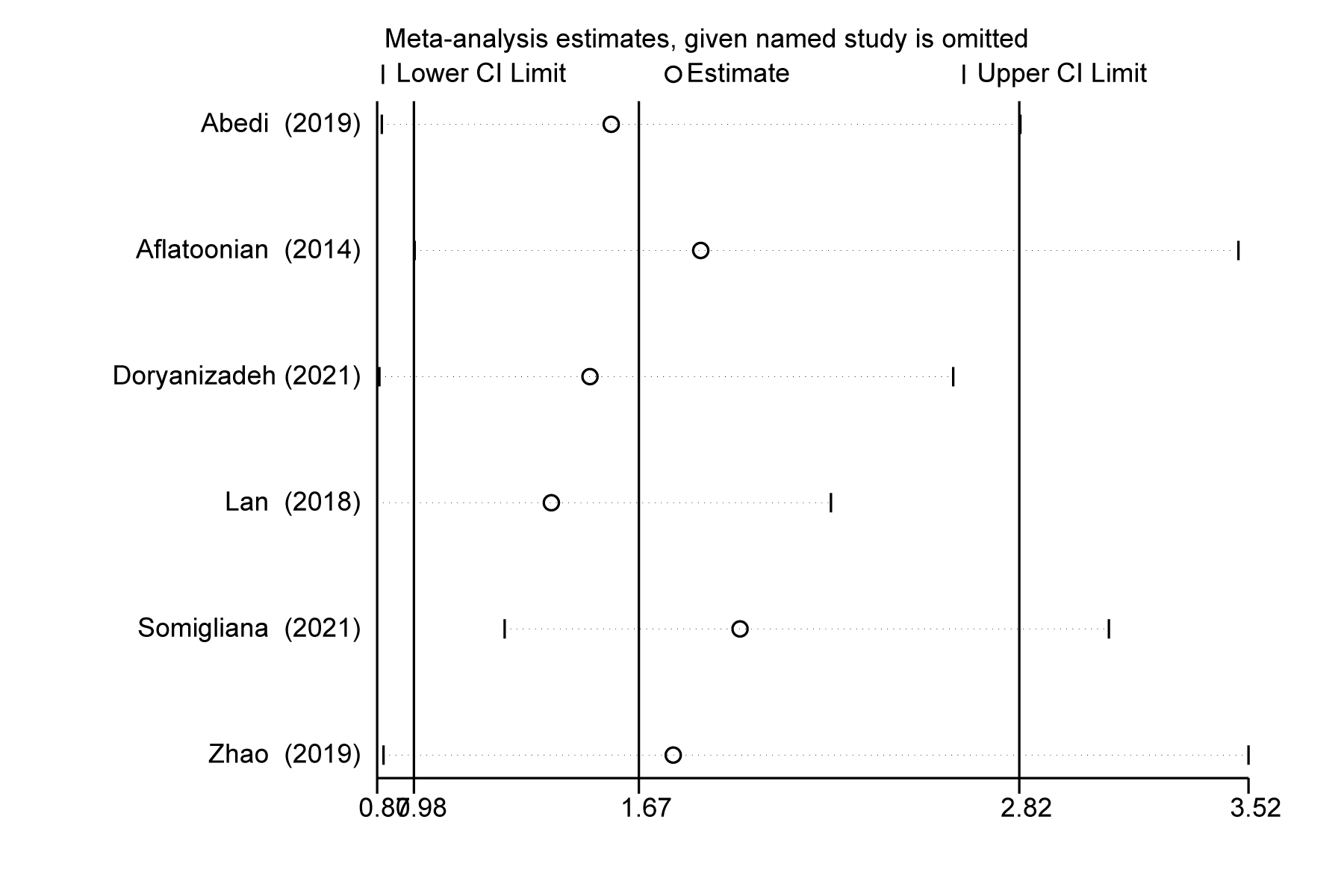

Supplement: Supplementary file 4 — Additional files 4: Fig. S3. Sensitivity analysis for the effect of vitamin D supplementation on the clinical pregnancy rate in studies of vitamin D only supplementation using random effect model (Odds Ratio). [file 12958_2023_1068_MOESM4_ESM.tif]

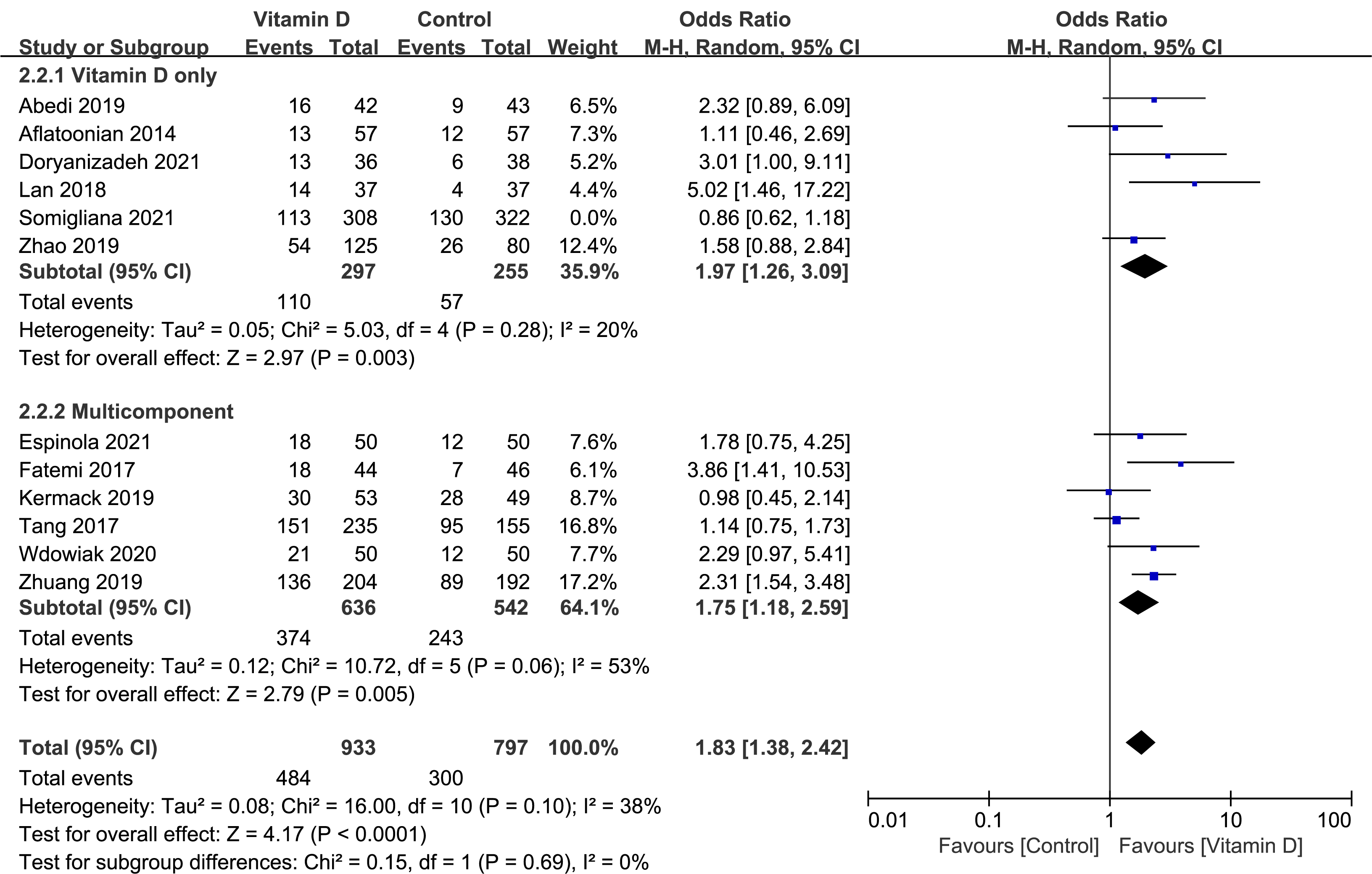

Supplement: Supplementary file 5 — Additional files 5: Fig. S4. Forrest plot for the effect of vitamin D supplementation on the clinical pregnancy rate in studies of vitamin D only supplementation [leave Somigliana (2021) out]. [file 12958_2023_1068_MOESM5_ESM.tif]
